# Supplementary material for: INTERMEDIUM-C mediates the shade-induced bud growth arrest in barley
Source: J Exp Bot. 2021 Dec 11;73(7):1963–77. doi: 10.1093/jxb/erab542 (PMC8982414; doi:10.1093/jxb/erab542)
Supplement: erab542_suppl_supplementary_figures_S1-S4 [file erab542_suppl_supplementary_figures_s1-s4.pdf]

Supplementary Information for *INTERMEDIUM-C* mediates the shade-induced bud growth arrest in barley

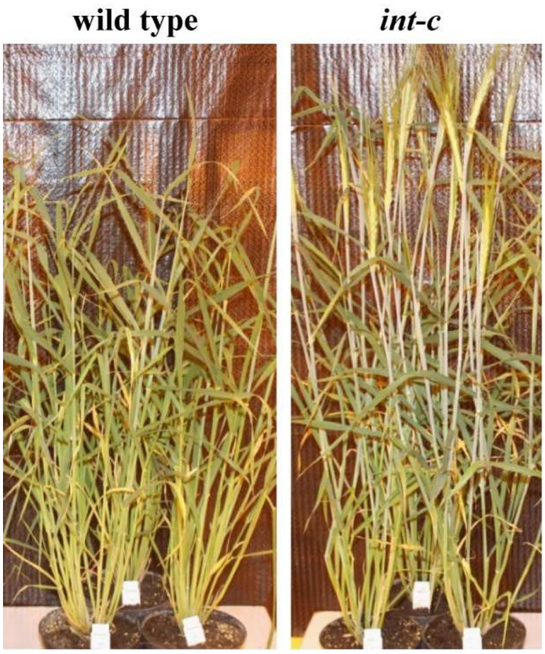

Supplementary Fig. S1. Flowering in wild type and *int-c* mutant plants at 15 weeks after germination. Shown are three independent replicates for each.

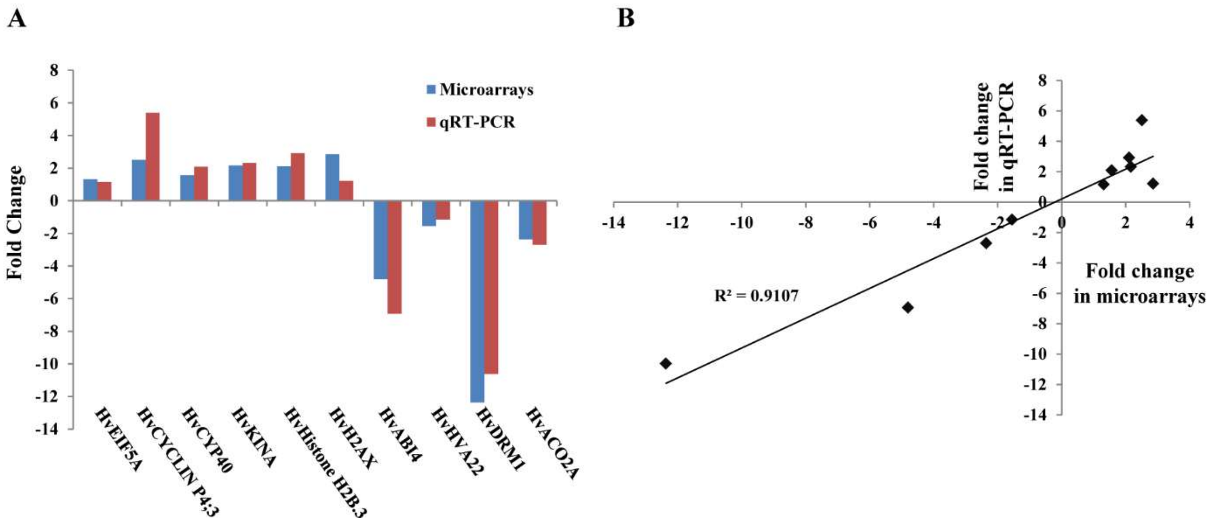

Supplementary Fig. S2. Validation of microarray data by qRT-PCR. A, mRNA levels of a subset of genes identified as responding to the decapitation treatment. B, Pearson's correlations between gene expression levels determined by qRT-PCR and microarray expression profiling for the same genes. The qRT-PCR and microarray data showed a very high average Pearson correlation coefficient (0.91), confirming the high reliability of the array data.

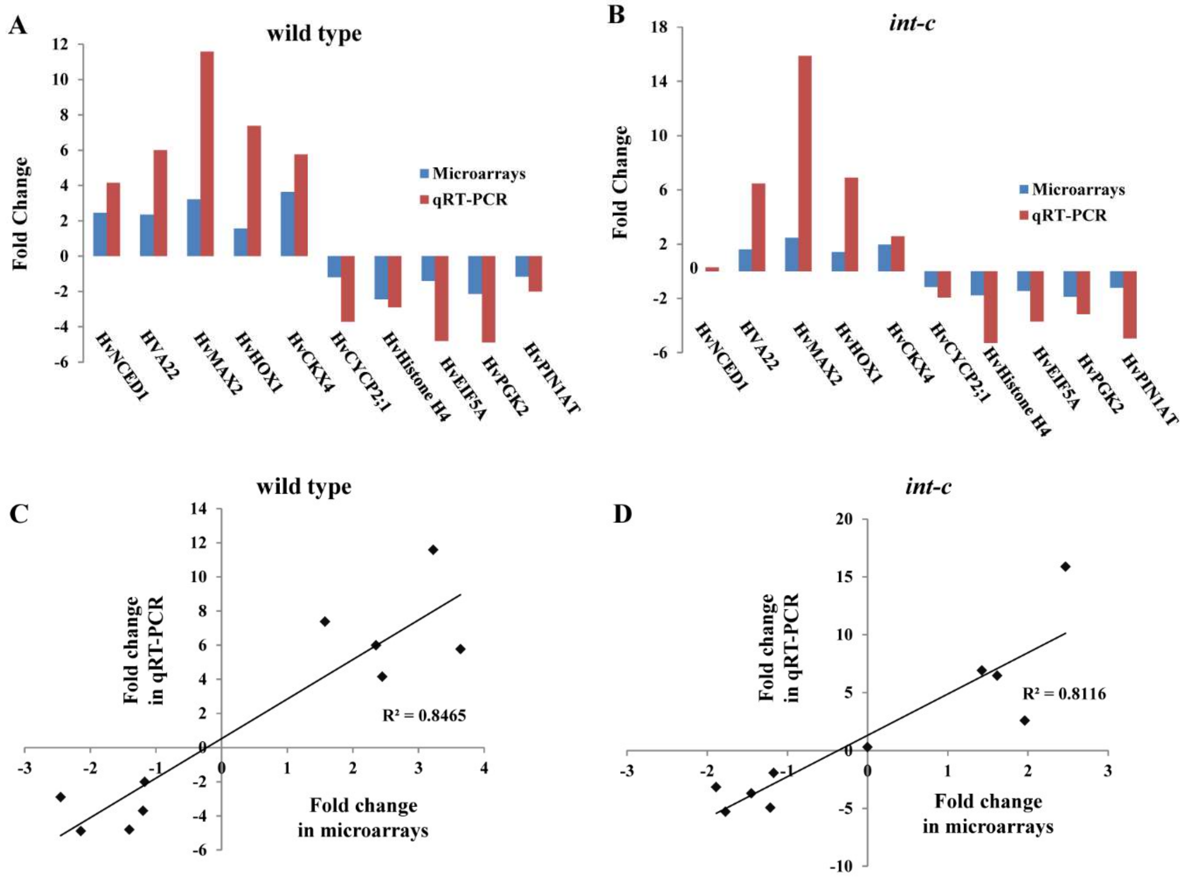

Supplementary Fig. S3. Validation of microarray data by qRT-PCR. A and B, mRNA levels of a subset of genes identified as responding to the shade treatment both in wild-type and *int-c* samples. C and D, Pearson's correlations between gene expression levels determined by qRT-PCR and microarray expression profiling for the same genes. Although the correlations of both datasets are high, microarray data underestimate the degree of change. The qRT-PCR and microarray data showed a very high average Pearson correlation coefficient (0.85 and 0.81 for the wild type and *int-c*, respectively), confirming the high reliability of the array data.

Supplementary Figure 4

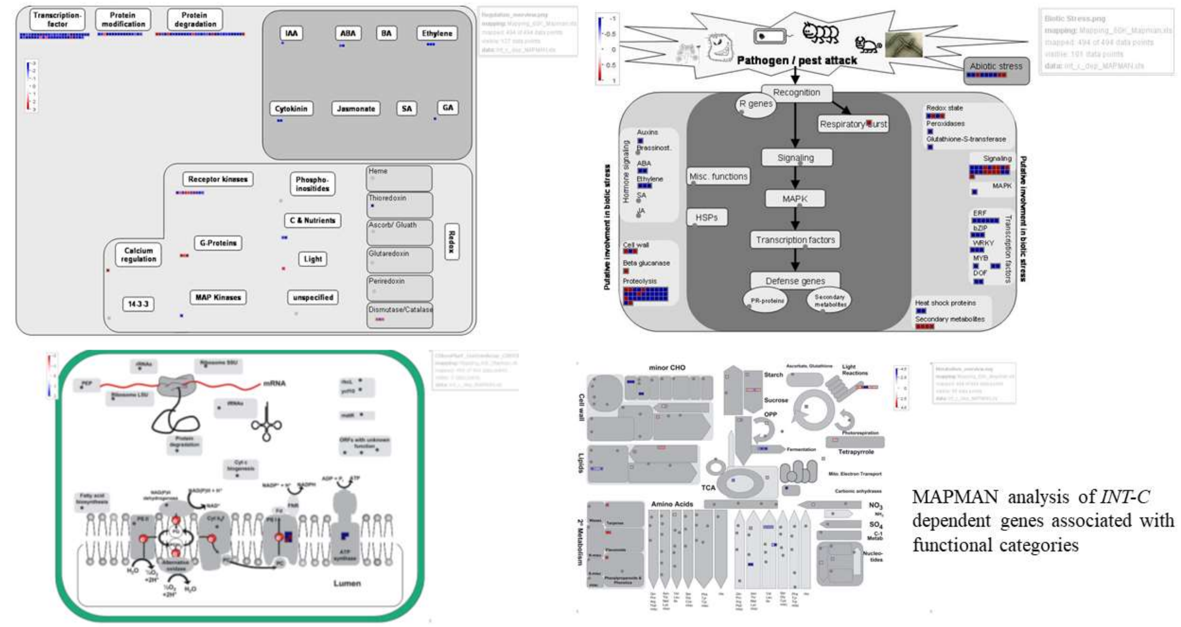

Supplementary Fig. S4. MAPMAN Analysis of DEGs for Regulation Overview, Biotic Stress, Chloroplast and Metabolism Overview.

Supplementary table 1: List of the primer sequences used in this study.
Supplementary table 2: List of TCP genes and associated gene expression derived from public databases.
Supplementary table 3: List of differential expressed genes (DEGs) for WT vs *int-c* (Control condition), WT vs *int-c* (shading condition), WT vs WT shading condition, *int-c* vs *int-c* shading condition, WT vs WT decapitation condition. Each with a list of associated direction of change.
Supplementary table 4: List of *INT-C* dependent genes, including comparison of direction after shading and decapitation.
